# Determining the Optimal Duration of Progesterone Supplementation prior to Transfer of Cryopreserved Embryos and Its Impact on Implantation and Pregnancy Rates: A Pilot Study

**DOI:** 10.1155/2016/7128485

**Published:** 2016-09-26

**Authors:** Sangita Sharma, Abha Majumdar

**Affiliations:** Sir Ganga Ram Hospital, Rajinder Nagar, New Delhi, India

## Abstract

*Objective.* To determine the optimal duration of progesterone supplementation prior to transfer of cryopreserved embryos and its impact on implantation and pregnancy rates.* Study Design*. Prospective randomised study.* Materials and Methods*. In an IVF unit of a tertiary centre, sixty-six patients undergoing cryopreserved embryo transfer cycles were included. Endometrial preparation was done with estradiol valerate. Once it reached a minimum of 7 mm, patients were allocated randomly into group I (*n* = 39) and group II (*n* = 27). Injectable progesterone 100 mg daily was then started for 3 and 4 days, respectively. This was followed by transfer of at least one thawed cleavage stage day 2 embryo of good quality. Groups I and II were compared in terms of clinical pregnancy and implantation rates.* Results*. In group I (3-day progesterone) and group II (4-day progesterone) the pregnancy rates were 41.02% (16/39) and 18.51% (5/27), respectively. On the other hand, the implantation rates were 16.82% (18/107) and 7.69% (6/78), respectively. The difference was statistically significant (*p* values 0.0172 and 0.0386, resp.).* Conclusion*. Progesterone supplementation for three days before the transfer of cleavage stage (day 2) cryopreserved embryos has significantly higher pregnancy and implantation rates, as compared to four-day supplementation.

## 1. Introduction

There is strong evidence that a temporal window of maximal endometrial receptivity exists in humans, corresponding with days 5–7 after ovulation. Endometrial receptivity consists of the acquisition of adhesion ligands with loss of inhibitory components which act as a barrier to the attaching embryo. This window of receptivity is determined by a large number of molecular mediators which are upregulated by progesterone levels. Theoretically it may be presumed that the number of days of exposure to progesterone will influence endometrial receptivity and hence implantation, although the endometrial exposure to estrogen during the follicular phase is equally important for progesterone to exhibit its maximal effect later. Inadequate uterine receptivity is responsible for approximately two-thirds of implantation failures in IVF cycles, whereas the embryo itself is responsible for only one-third of these failures [1, 2].

There is a wealth of data on the dosage and modes of administration of progesterone after embryo transfer as luteal phase support and little work has also been done on the dosage and modes of progesterone supplementation prior to embryo transfer, but there is scarcity of data on the optimal duration of progesterone supplementation prior to embryo transfer and its effect on implantation and pregnancy rates [3–8].

The optimal duration of progesterone supplementation and the development of maximal endometrial receptivity that is the implantation window can be studied either by taking endometrial biopsies and evaluating for the presence of pinopodes (under scanning electron microscopy) and other biomarkers of implantation (e.g., the expression of @Vb3, PP14, and HOXA 10 gene expression) or by transferring the embryos and observing the pregnancy and implantation rates. The former method is not possible in transfer cycles and moreover the facilities might not be available in most of the centres.

This study is being conducted with the aim to determine whether the number of days of progesterone exposure prior to the transfer of cryopreserved embryos will influence implantation and pregnancy rates, and if it would, then what is the optimal duration of progesterone supplementation prior to embryo transfer?

Cryopreserved embryo transfer cycles are chosen to be studied, as it appears to be the best human model for determining the optimal duration of progesterone supplementation, where the number of days of progesterone exposure can be modified, keeping the stage of embryo at transfer constant. The cryopreserved embryo transfer cycles will include

- surplus embryos for use in subsequent cycle,
- embryos from donor-recipient cycles.

## 2. Aims and Objectives

The objectives are to determine the optimal duration of progesterone supplementation prior to transfer of cryopreserved embryos and its impact on implantation and pregnancy rates.

## 3. Material and Method

### 3.1. Study Design

It is a prospective randomized study.

The study was conducted at the IVF and human reproduction unit of a tertiary care centre in India, over a period of 9 months.

### 3.2. Study Population

**Figure 1:**
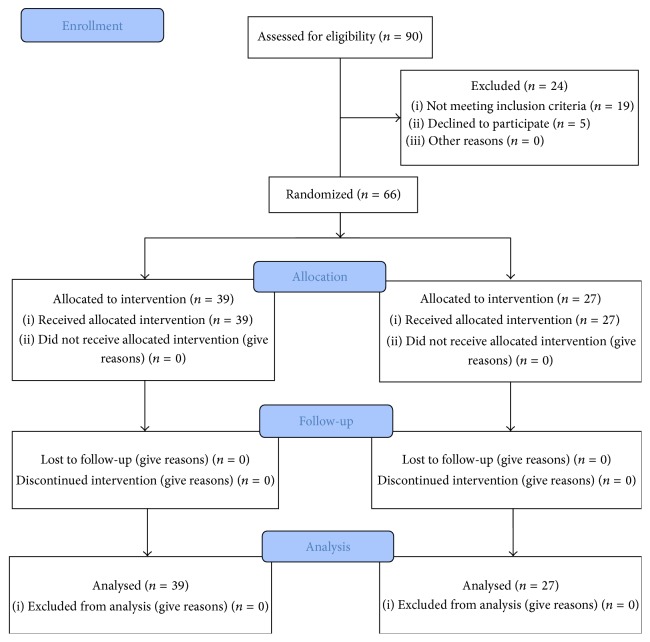
CONSORT 2010 flow diagram.

Sixty-six Indian women undergoing cryopreserved embryo transfer cycles were included in the study after considering the inclusion and exclusion criteria (Figure 1). Proper consent was taken from the patients and approval was taken from the institutional review board.

 Inclusion criteria are as follows:

- All embryos cryopreserved from women <37 yrs of age.
- Cryopreserved cleavage stage embryos.
- Endometrial thickness on day of starting progesterone >7 mm.
- Transfer of at least 1 postthaw fully intact embryo.

Exclusion criteria are as follows:

- Age of patient (and of oocyte donor) >37 years.
- Natural cycles with no estrogen supplementation.
- Endometrial thickness on day of starting progesterone <7 mm.
- Adenomyosis or intramural fibroid >4 cm.
- Demonstrable hydrosalpinx.
- History of 3 previous unsuccessful IVF cycles.

A transvaginal scan was performed on third day of the menstrual cycle, along with basal FSH, LH, and estradiol levels. Confirming the baseline endometrium to be less than 4 mm and estradiol levels less than 50 pg/mL, artificial preparation of endometrium was started with estradiol valerate 2 mg thrice daily for 12 days, after which a transvaginal scan was repeated to see the endometrial thickness and pattern. If the endometrial thickness reached a minimum of 7 mm, the patients were randomly allocated to group I and group II. If the ET was found to be less than 7 mm, estradiol valerate was continued for another 2-3 days and reassessed. It was planned initially that if still the endometrial thickness would remain suboptimal, the patients would be excluded from the study, but fortunately, in all the patients, it reached a minimum of 7 mm. Once the endometrium reached a minimum of 7 mm, patients were then allocated randomly into group I (*n* = 39) and group II (*n* = 27) by a nurse who assigned participants to their groups (Figure 1). The randomization was done on the basis of a computer generated randomization table. The team performing the embryo transfer was blinded to group assignment. Trial was not placebo controlled as the outcome measures were objective. The patients were then started on injectable progesterone 100 mg daily, for 3 and 4 days, respectively.

*Group I*. In this group, injectable progesterone was given for 3 days and ET was done on 4th day.

*Group II*. In this group, injectable progesterone was given for 4 days and ET was done on 5th day.

The cryopreserved embryos were thawed on the day of the embryo transfer. After being thawed, the embryos were examined, and the cycle was included in the study if at least one embryo of good quality [24] was found to be fully intact.

This was followed by embryo transfer of at least one postthaw fully intact cleavage stage (day 2) embryo of good quality. Luteal support was given in the form of micronised progesterone 800 mg intravaginally in two divided doses, along with estradiol valerate 2 mg thrice daily. All patients were tested for pregnancy after 14 days of embryo transfer by checking serum bhCG levels. On confirmation of pregnancy, luteal support was continued till 14 weeks. A transvaginal scan was done 4 weeks after the embryo transfer to see for a gestational sac and confirm a clinical pregnancy. Comparative analysis of the clinical pregnancy and implantation rates in groups I and II was done. 

*Outcome Measures*

- Primary outcome is as follows:
  - Clinical pregnancy rate.
  - Implantation rates.

Clinical pregnancy rate (CPR) was calculated separately for each group as the number of patients who became pregnant (confirmed by the presence of gestational sac on tranvaginal scan 4 weeks after embryo transfer) divided by the number of patients who underwent embryo transfer. Implantation rates (IR) were also calculated for each group, as the number of gestational sacs divided by the number of embryos transferred.

### 3.3. Statistical Analysis

The outcome of the treatment cycles in terms of pregnancy and implantation rates was compared using Student's *t*-test. *p* value < 0.05 was considered to be statistically significant.

## 4. Results

A total of 66 patients were enrolled in the study, 39 in group I and 27 in group II.

There was no significant difference in both groups with regard to age. The patients in both groups were comparable in terms of days of estrogen exposure, endometrial thickness at the time of embryo transfer, and the number of embryos transferred (Table 1).

**Figure 2:**
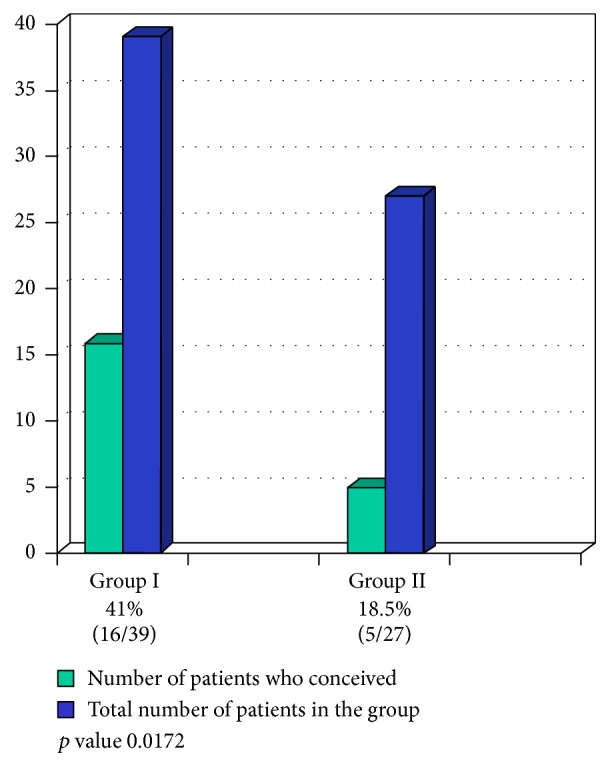
Pregnancy rates in patients receiving progesterone for 3 and 4 days before embryo transfer.

**Figure 3:**
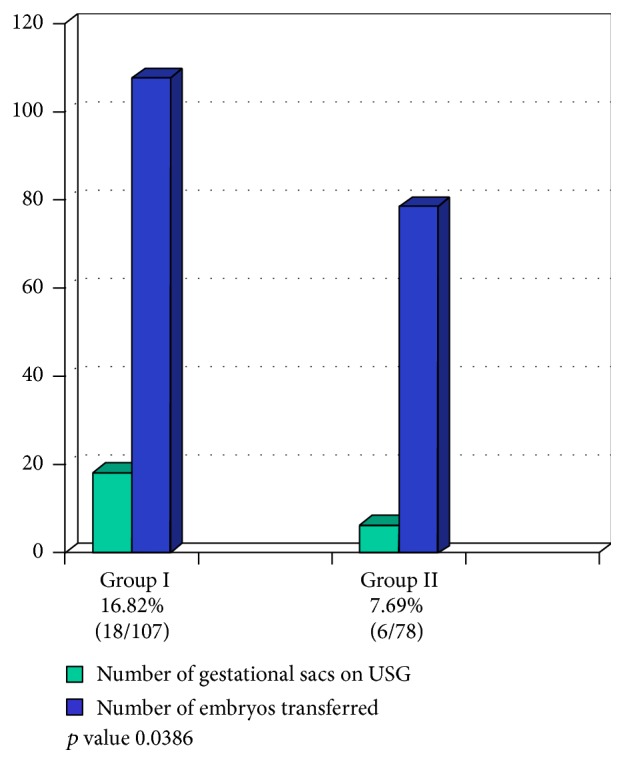
Implantation rates in patients receiving progesterone for 3 and 4 days before embryo transfer.

In group I (3 days of progesterone exposure) and group II (4 days of progesterone exposure) the pregnancy rates were 41.02% (16/39) and 18.51% (5/27), respectively. On the other hand the implantation rates were 16.82% (18/107) and 7.69% (6/78), respectively, whereas the multiple pregnancy rate was 12.5% (2/16) and 20% (1/5) for groups I and II. The difference was statistically significant for both the pregnancy (*p* value 0.0172) and implantation rates (*p* value 0.0386) (Figures 2 and 3).

There was such a significant difference in pregnancy and implantation rates in both groups that the study had to be aborted prematurely before reaching the predecided target of 100 patients.

## 5. Discussion

For successful implantation to occur, a viable embryo has to meet the endometrium in the right phase of receptivity, known as the implantation window. Detection of pinopods as a marker of uterine receptivity has been reported in the past [25–27]. Expression of certain genes which signal cellular adhesion pathways is essential by the endometrium for implantation. Progesterone exposure is responsible for the changes in estrogen primed endometrium, which makes it receptive for implantation of an embryo. Implantation window in humans is known to begin after 5–7 days of ovulation and remains open for another 4-5 days. That means maximal endometrial receptivity in a natural 28-day menstrual cycle is from day 19 to day 24.

In most IVF clinics worldwide, the practice is to supplement progesterone for 3 days before transferring a cryopreserved day 3 embryo and for 5 days before transferring a cryopreserved day 5 blastocyst. So, the number of days of progesterone exposure before embryo transfer depends on the stage of the frozen embryos to be transferred. The logic is to bring the endometrium to the same level of maturity as it would have been during natural implantation. But there are no randomized controlled trials in our knowledge to support this logic based practice. Moreover, a small concern here is that, in a natural cycle, some amount of progesterone synthesis begins after the LH surge, even before ovulation. Similarly, in a fresh IVF cycle, the progesterone starts increasing after the hCG trigger. Therefore it seems that, in a frozen thawed embryo transfer cycle, the endometrium lags behind in terms of maturity and progesterone exposure if the progesterone is supplemented for the same number of days as is the stage of the embryo being transferred. It was also mentioned by Navot et al. in 1986 that during a normal implantation, a 4- to 8-cell stage embryo coincides with endometrial development 3-4 days after the LH surge* in vivo* [28].

To support this, a few, rather a couple of studies, have shown that pregnancy rates were better when progesterone was supplemented for 4 or 5 days before transferring cleavage stage (4–8-cell stage) embryos [28]. Prapas et al. in 1998 also reported a higher pregnancy (40%) and implantation rate (14.1%) when progesterone was supplemented for 4 days as compared to 3 days prior to day 2 stage of embryo transfers [5]. They studied the implantation window in oocyte donation cycles, depending on the duration of progesterone therapy [5] and found that implantation and pregnancy rates were significantly higher after progesterone administration for 4 and 5 days (40% and 48.3%, resp.), as compared to 0%, 12%, and 20.4% after administration of progesterone for 2, 3, and 6 days, respectively. All transfers were performed within 48 hours of insemination (day 2 embryo stage). They found that progesterone exposure should be for a minimum of 48 hours for implantation to occur.

On the contrary, there are another couple of studies, which show that the pregnancy and implantation rates are better if the days of exposure of progesterone coincide with the stage of the embryo transferred. Ding et al. in 2007 studied 49 frozen thawed blastocyst transfer cycles and found that clinical pregnancy rates, ongoing pregnancy, and implantation rates were higher when progesterone was given for 5 days before the transfer as compared to 6 days (60.9% versus 53.8%, 56.5% versus 50.0%, and 40.7% versus 30.0%, resp.), but the differences did not reach statistical significance (*p*s > 0.05). However, this study included thawed blastocyst transfer cycles, when there is a critical margin before the implantation window closes and one extra day of progesterone supplementation might affect the results adversely [7].

Still, a few more authors noted no difference in pregnancy or implantation rates with 3, 4, or 5 days of progesterone supplementation prior to embryo transfer. In a prospective study by Navot et al. (1991) 60 recipients of oocyte donation programme were studied and embryo transfer (day 2 or 3) after 1,2, 3,4, 5, and 6 days of progesterone administration had no significant effect on pregnancy and abortion rates [3]. It was shown by Michalas et al. in 1996 that the variation in progesterone administration between 2 and 4 days before embryo transfer (day 2) did not affect pregnancy outcome [4].

Recently a retrospective analysis of 1103 frozen thaw embryo transfer cycles was published in Chinese, where pregnancy rates were studied for 3 and 4 days of progesterone administration followed by transfer of day 3 embryos [8]. They also studied the outcome of the frozen thaw cycles where day 5 blastocysts were transferred after 5 and 6 days of progesterone administration. They found that the implantation rate, pregnancy rate, ectopic pregnancy rate, multiple pregnancy rate, and early abortion rate were not significantly different when day 3 embryos were transferred after 3 or 4 days of progesterone and also when day 5 blastocysts were transferred after 5 or 6 days of progesterone. Interestingly, 3 or 4 days of progesterone supplementation for day 5 blastocyst transfers or 5 or 6 days of progesterone before day 3 embryo transfers was not studied, probably because our logic based practice over the years and satisfactory results have cleared some doubts on their own.

Table 2 shows some studies dealing with estrogen/progesterone supplementation to prepare endometrium before transfer of cryopreserved thawed embryos. The point to be emphasized here is that, even before day 2 embryo transfers, most of the centres prefer to administer progesterone for at least 3 days rather than 2. However, for day 3 and day 5 transfers, the number of days of progesterone administration was the same as that of the stage of the embryos transferred. This is in accordance with the study of Prapas et al. [5], where they concluded that at least 48 hours of progesterone exposure is essential to open the implantation window.

However, till date there is no prospective randomized controlled trial, in our knowledge, to address the issue of the optimal duration of progesterone administration prior to transfer of cryopreserved embryos and its impact on implantation and pregnancy rates.

In our study, we found that duration of progesterone supplementation is critical for implantation. In this study, progesterone supplementation for three days before the transfer of cryopreserved cleavage stage embryos (day 2; 4–6-cell stage) had significantly higher pregnancy and implantation rates, as compared to 4 days of progesterone administration. Moreover, before the commencement of this study, our practice has been to transfer thawed day 2 embryos after 2 or 3 days of progesterone exposure in a cryopreserved embryo transfer cycle, the results being satisfactory and comparable. Thus we conclude that pregnancy and implantation rates are better if the number of days of progesterone is the same or one day more than the stage of the cryo-thawed embryo being transferred. But a difference of two days might become detrimental to the results. Large multicentric, randomised controlled studies are required before the exact duration of progesterone supplementation can be decided.

A prospective randomized trial is being conducted at the Centre of Reproductive Medicine of the Brussels University Hospital, by the official title “Optimal Length of Progesterone Supplementation Before the Transfer of Cryopreserved (Frozen)-Thawed Day 3 Embryos in an Artificial Cycle With Exogenous Estrogen and Progesterone (PROFETA-3)” (ClinicalTrials.gov identifier NCT01940653), where they are comparing their practice of starting progesterone supplementation 5 days before the transfer of a day 3 embryo, with the more common practice at most of the centres to start it 3 days before a day 3 transfer [29].

Similarly, another trial is going on (PROFETA-5)** (**ClinicalTrials.gov identifier NCT02032797), at the same centre, where again they are comparing their practice of starting progesterone supplementation 7 days before the transfer of a day 5 blastocyst, with the more common practice at most of the centres to start it 5 days before [30]. We hope to get the results ready by the end of 2016.

## 6. Conclusion

Progesterone supplementation for three days before the transfer of cleavage stage (day 2) cryopreserved embryos has significantly higher pregnancy and implantation rates, as compared to four-day supplementation.

## Additional Points

*Capsule*. In a prospective study, it was found that progesterone supplementation for three days before the transfer of cleavage stage cryopreserved embryos has significantly higher pregnancy and implantation rates, as compared to four-day supplementation.

## Figures and Tables

**Table 1:** Comparison of characteristics of the patients receiving progesterone for 3 and 4 days before embryo transfer.

Variables	Group I (3 days of P) (*n* = 39)	Group II (4 days of P) (*n* = 27)	*p* value
Age (yrs)	30.24 + 4.7	29.86 + 4.4	NS
Days of estrogen exposure	12.3	13.2	NS
Endometrial thickness (mm)	8.42 + 1.41	8.64 + 1.22	NS
Number of embryos transferred	2.72 + 0.73	2.77 + 0.74	NS

**Table 2:** Some studies dealing with estrogen/progesterone supplementation to prepare endometrium before transfer of cryopreserved-thawed embryos.

Reference	Transfer of the following:	Estrogen preparation by the following:	Progesterone preparation by the following:	Days of progesterone exposure
Muasher et al., 1991 [9]	Day 2 embryos	Estradiol patches	Intramuscular progesterone	3
Pattinson et al., 1992 [10]	Day 2 embryos	Estradiol	Vaginal progesterone	3
Pattinson et al., 1994 [11]	Day 2 embryos	Estradiol	Vaginal progesterone	3
Lelaidier et al., 1995 [12]	Blastocysts	Estradiol	Vaginal progesterone	5
Queenan et al., 1997 [13]	Day 2 embryos	Estradiol patches	Intramuscular progesterone	3
Queenan et al., 1997 [14]	Day 2 embryos	Estradiol patches	Intramuscular progesterone	3
Horne et al., 1997 [15]	Day 2 embryos	Estradiol valerate	Vaginal progesterone	4
Simon et al., 1998 [16]	Day 2-3 embryos	Estradiol	Vaginal progesterone	2-3
Simon et al., 1999 [17]	Day 2-3 embryos	Estradiol	Vaginal progesterone	2-3
Banz et al., 2002 [18]	Day 2 embryos	Estradiol patches	Vaginal progesterone	3
Seelig et al., 2002 [19]	Day 2 embryos	Estradiol valerate	Vaginal progesterone	3
Schröder et al., 2003 [20]	Day 2 embryos	Estradiol patches	Vaginal progesterone	3
Dal Prato et al., 2002 [21]	Day 2 embryos	Estradiol patches	Intramuscular progesterone	3
Boldt et al., 2003 [22]	Day 3 embryos	Estradiol	Intramuscular progesterone	3
Revel et al., 2004 [23]	Day 3 embryos	Estradiol	Vaginal progesterone	3

Source: Nawroth and Ludwig [6].
